# Prevalence of metabolic syndrome among HIV-positive and HIV-negative populations in sub-Saharan Africa—a systematic review and meta-analysis

**DOI:** 10.1186/s13643-018-0927-y

**Published:** 2019-01-03

**Authors:** Olamide O. Todowede, Solange Z. Mianda, Benn Sartorius

**Affiliations:** 1 Department of Public Health Medicine, School of Nursing and Public Health, University of KwaZulu-Natal, Durban, 4001 South Africa; 2 Department of Health Metrics Sciences, School of Medicine, University of Washington, Seattle, USA

**Keywords:** Metabolic syndrome, Sub-Saharan Africa, HIV-negative, HIV-positive

## Abstract

**Background:**

Metabolic syndrome (MetS) is a constellation of conditions that increase the risk of cardiovascular diseases. It is an emerging concern in sub-Saharan African (SSA) countries, particularly because of an increasingly aging population and lifestyle changes. There is an increased risk of MetS and its components among people living with Human immune deficiency syndrome (HIV) individuals; however, the prevalence of metabolic syndrome in the SSA population and its differential contribution by HIV status is not yet established. This systematic review and meta-analysis were conducted to estimate the pooled prevalence of metabolic syndrome in people living with HIV and uninfected populations, its variation by sub-components.

**Methods:**

We performed a comprehensive search on major databases—MEDLINE (PubMed), EBSCOhost, and Cochrane Database of Systematic Reviews and Web of sciences for original epidemiological research articles that compared proportions of the MetS and its subcomponents between people living with HIV and uninfected patients and published between January 1990–December 2017. The inclusion criteria were adults aged ≥ 18 years, with confirmed HIV status. We assessed the risk of bias using a prevalence studies tool, and random effect meta-analyses were used to compute the pooled overall prevalence.

**Results:**

A total of four cross-sectional studies comprising 496 HIV uninfected and 731 infected participants were included in the meta-analysis. The overall prevalence of MetS among people living with HIV was 21.5% (95% CI 15.09–26.86) versus uninfected 12.0% (95% CI 5.00–21.00%), with substantial heterogeneity. The reported relative risk estimate for MetS among the two groups was twofold (RR 1.83, 95% CI 0.98–3.41), with an estimated predictive interval of 0.15 to 22.43 and *P* = 0.055 higher for the infected population. Hypertension was the most prevalent MetS sub-components, with diverse proportions of people living with HIV (5.2–50.0%) and uninfected (10.0–59.0%) populations.

**Conclusions:**

The high range of MetS prevalence in the HIV-infected population compared to the uninfected population highlights the possible presence of HIV related drivers of MetS. Also, the reported high rate of MetS, irrespective of HIV status, indicates a major metabolic disorder epidemic that requires urgent prevention and management programs in SSA. Similarly, in the era of universal test and treat strategy among people living with HIV cohorts, routine check-up of MetS sub-components is required in HIV management as biomarkers.

**Systematic review registration:**

PROSPERO CRD42016045727

## Introduction

The problem of metabolic syndrome (MetS) has been the main scourge of high mortality and morbidity [1]. Globally, the prevalence of metabolic syndrome (MetS) is unknown [2], and country-specific prevalence varies with estimated prevalences in excess of 25.0% in developed countries [3–5]. In sub-Saharan Africa (SSA), the prevalence of metabolic syndrome is not well established. The growing burden of the global metabolic disorder is occurring at a time when SSA is experiencing an epidemiological transition, whereby the continent is affected by the dual burden of infectious and non-communicable diseases [6]. Further, SSA remains the worst affected region globally with over 25 million people living with HIV [7]. The coexistence of infectious diseases and non-communicable diseases is well documented in developed countries, and the intensity of this comorbidity is incomparable in SSA [8].

The global response to HIV has averted 30 million new infections and nearly 8 million AIDS-related deaths, as a result of antiretroviral therapy (ART) uptake [9]. This has resulted in an aging population of people living with HIV, living longer on ART and at greater risk of chronic diseases and metabolic disorders [10, 11]. While the global focus is on preventing and managing HIV infections, less attention is on the metabolic impact of HIV infection and treatment on infected individuals. The global pooled prevalence of metabolic risk factors among people living with HIV range from 16.7 to 31.3% [12].

Increased risk of MetS and its subcomponents among people living with HIV individuals is well documented and attributed to HIV infection, antiretroviral therapy, and other related factors [13, 14]. Of which is similar to the burden of MetS risk in the general population, as a result of associated modifiable risk factors [15, 16]. However, little is known about the pooled prevalence of MetS and the prevalence difference among people living with HIV and uninfected population in SSA. Studies from developed countries predominantly report MetS among HIV-positive cohorts than in negative counterparts [15–17]. Studies suggest that MetS outcomes are much lower among people living with HIV compared to the general population, but ART-treated patients have a higher risk of metabolic complication [18]. Whether this risk is more or less among people infected with HIV compared to uninfected population remains controversial. This systematic review and meta-analysis was conducted to understand the burden of metabolic syndrome and its subcomponents among people living with HIV and uninfected population in SSA.

## Methods

### Outcome of interest

The primary outcome of this study was to compare the pooled prevalence of metabolic syndrome among people living with HIV and uninfected populations in SSA. The secondary aim was to compare metabolic syndrome subcomponents (namely visceral obesity, hypertension, diabetes, triglycerides, HDL cholesterol) among people living with HIV and uninfected populations in SSA.

### Protocol and registration

A study protocol (published) was developed prior to the conduct of this review [19]. The protocol was registered in the PROSPERO international prospective register of systematic reviews (CRD42016045727). The protocol was amended by removing the aspect of co-morbid diabetes and hypertension components. The Preferred Reporting Items for Systematic Reviews and Meta-Analysis (PRISMA) guidelines were followed [20, 21].

### Eligibility criteria

All studies (randomized control trials, cross-sectional, case-control, and cohort studies) among adults (18+) published from January 1990–December 2017 reporting the prevalence of metabolic syndrome and its subcomponents in people living with HIV and/or uninfected populations were considered for inclusion. We excluded studies that presented estimates from study participants with unknown HIV status. The full inclusion and exclusion criteria are presented in the aforementioned published protocol [19].

### Search strategy and selection process

**Fig. 1:**
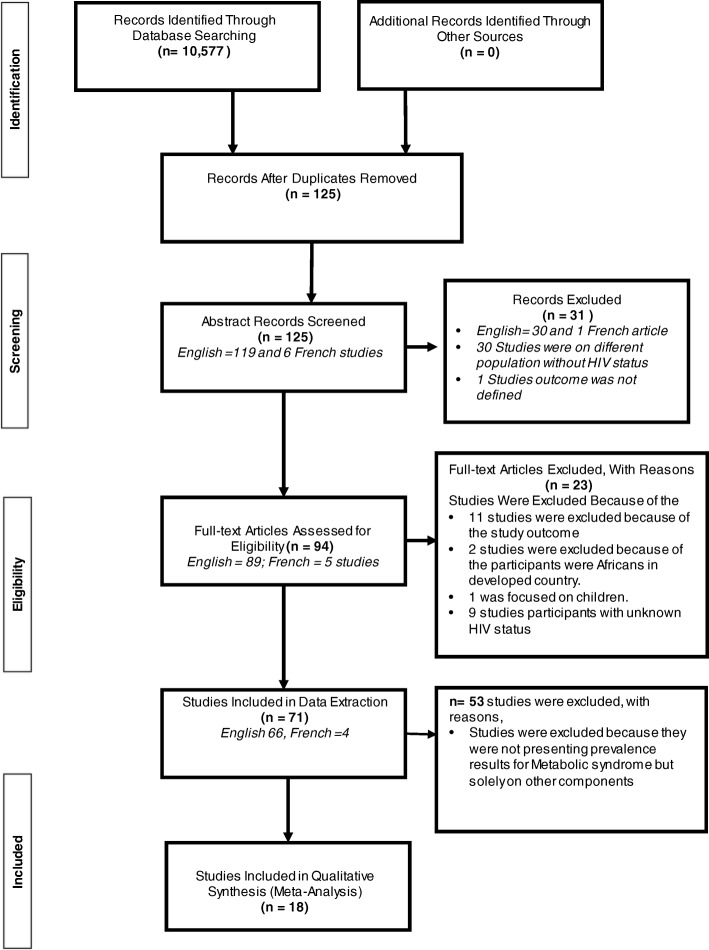
PRISMA flow diagram of study selection process

MEDLINE (PubMed), EBSCOhost, and Cochrane Database of Systematic Reviews and Web of sciences databases were searched for papers published between January 1990 and December 2017. The search terms used a combination of relevant medical subject headings (MeSH) and database specific terms with an African search filter. The search strategy and the number of returned items are presented in Appendix 1 and 2. The reference lists of identified articles were traced through a web of science, and conference proceedings checked using the International AIDS society abstract archives. The titles of retrieved articles were examined to exclude ineligible articles. Given a large number of Francophile countries in SSA, this review included eligible studies published in French and were reviewed by a French-speaking reviewer (SM). The selection of studies was a multistep process with two reviewers (OO, SM) independently screening the abstract and full text for potential eligibility using the inclusion criteria, and discrepancies were resolved through arbitration with a third reviewer (BS). The flow diagram of the study selection and exclusion process is presented in Fig. 1. The interrater agreement for abstract and full-text screening was high at 90% and 100% respectively.

### Assessment of the methodological quality and risk of bias of included studies

The methodological quality and risk of bias of selected articles were assessed using the Effective Public Health Practice Project/McMaster Evidence Review and Synthesis Centre Tool: Quality Assessment Tool for Quantitative Studies [22], and the risk of bias tool for prevalence studies [23]. A multistep process approach was employed by two reviewers (OO, SM), and the interrater agreement on quality and risk of bias were 80% and 90% respectively. A summary of the areas considered in the assessment of each domain is included in Appendix 1 and 2—the risk of bias and quality assessment of included studies.

### Data item and collection process

Abstraction of meta-data from the included articles was performed using a specifically designed Google form, by two reviewers (OO and SM) independently. Information extracted included publication details, population sampled and sample size, metabolic prevalence estimates, participant’s characteristics, and HIV status. Prevalence figures and 95% confidence intervals (CIs) were extracted or calculated from the available data using the Clopper-Pearson method [24]. Certain authors were contacted for clarifications and/or further data requests, and if contacted three times and no response, the article concerned was removed from the included studies.

### Analysis

Data were analyzed using Stata 13.0 (StataCorp. 2013. Stata Statistical Software: Release 13. College Station, TX: StataCorp LP). Heterogeneity between study estimates was assessed using the *I*^2^ statistic, i.e., the percentage of variation not due to sampling error across studies. An *I*^2^ value above 50% indicates high heterogeneity. The meta-weighted prevalence (95% confidence interval (CI)) of metabolic syndrome among HIV-positive and HIV-negative populations was undertaken using a random effects model (to account for heterogeneity). For the studies with both HIV-positive and control HIV-negative population, we estimated a meta-weighted relative risk using a random effects approach. The influence of the estimates for all included studies was assessed by the level of risk of bias, quality of the study, study settings, and data collection methods. The risk of bias and study quality were classified as either low, moderate, or high, and we performed the Egger test (in addition to funnel plot for the HIV-positive only meta-analysis) to assess for potential publication/small-study bias. The association between HIV infection and MetS was estimated directly using the “metan” function in STATA, where the default is RR, i.e., pooled risk ratio. We also check for influential outlying studies using a random effects variance shift outlier model (RVSOM) for detecting and accommodating outliers in a meta-analysis [25].

In a meta-analysis of prevalence, when the estimate for a given study tends towards either 0.0% or 100.0%, the variance for that study moves towards zero, and as a result, its relative weight may be overestimated [26]. Thus, we transformed the prevalence estimates using the double arcsine method to correct for this potential discontinuity [26, 27]. For data analysis, we merged the estimates of people living with HIV population by ATP III, IDF, JIS, and WHO definitions criteria (average prevalence across those with multiple definitions). The final pooled meta-estimates and 95% CIs were back-transformed for ease of interpretation. We were unable to stratify prevalence estimates by age, sex, and location with sufficient power, due to the limited number of studies (four) that compared estimates between HIV-negative and HIV-positive and resultant sample sizes.

## Results

### Included studies search process

Our search returned a total of 10,577 publications, and the titles were screened for eligibility and duplicates were removed. A total of 125 articles were eligible for abstracts screening. Based on the abstract screening, 94 articles were eligible for full-text screening and 31 articles were excluded. Among the 94 articles that were reviewed full-text, 18 articles met the inclusion criteria, contained or allowed the estimation of MetS prevalence estimates, and were selected for inclusion in this review [28–45].

### Characteristics of included studies

#### Study participation

The characteristics of the included studies are summarized in Table 1. Of the 18 studies included in this review, 4 studies [33, 35, 38, 40] compared the prevalence of MetS among people living with HIV and uninfected populations while the other 14 presented MetS prevalence estimates among people living with HIV subjects only [28–32, 34, 36, 37, 39, 41–45].

**Table 1:** Characteristics of included studies

	Author and publication year	Study design, settings, and year	Sex	Mean age (years)	Matched mean age (years)		HIV status		Hypertension definition criteria used for MetS estimate	MetS definition criteria
					HIV+	HIV−	HIV+	HIV−		
1	Amusa et al., 2016 [33]	Cross sectional, Nigeria, NS	Both	41 ± 7/40 ± 8 ^α^	41 ± 7	40 ± 8	150	50	Not stated	Other
2	Ayodele et al., 2012 [32]	Cross sectional, Nigeria, NS	Both	39.5–9.3	NA	NA	291	NA	≥ 130/85 and on antihypertensive treatment	IDF, ATP, JIS
3	Berhane et al., 2012 [34]	Cross-sectional, Ethiopia, 2010	Both	18 and above	NA	NA	313	NA	≥140/90 and on antihypertensive treatment	ATP
4	Tesfaye et al., 2014 [42]	Cross sectional, Ethiopia, 2012–13	Both	32.7 ± 9.7 (ART) 32.6 ± 7.8 (naïve)	NA	NA	374	NA	≥ 130/85 and on antihypertensive treatment	IDF, ATP
5	Sobieszczyk et al., 2016 [29]	Cross-sectional, South Africa, 2013	Female	Median 24 years	NA	NA	160	NA	≥ 130/85 and on antihypertensive treatment	ATP
6	Obirikorang et al., 2016 [41]	Cross sectional, Ghana,2013	Both	40.3 ± 0.8	NA	NA	433	NA	≥ 130/85 and on antihypertensive treatment	IDF, ATP, WHO
7	Ngatchou et al., 2013 [40]	Cross sectional, Cameroon, 2009–10	Both	41 ± 12^α^/39 ± 10	39.0 ± 10.0	41 ± 12	108	96	≥ 140/90 and on antihypertensive treatment	IDF
8	Fourie et al., 2010 [35]	Case control, South Africa, 2005	Both	44 ± 7.81^α^/44 ± 8.04	44.0 ± 8.04	44.0 ± 7.81	300	300	≥ 130/85 and on antihypertensive treatment	IDF, ATP
9	Muhammad et al., 2013 [45]	Cross sectional, Nigeria, 2009	Both	32.5 ± 7.55	NA	NA	200	NA	≥ 140/90 and on antihypertensive treatment	IDF
10	Mbunkah et al., 2014 [38]	Cross sectional, Cameroon, 2010–11	Both	18–70	41.1 ± 11.2	47.3 ± 13.7	173	50	≥ 130/85 and on antihypertensive treatment	ATP
11	Guehi et al., 2016 [30]	Randomized control trial, Ivory Coast, 2008–14	Both	29–42	NA	NA	755	NA	≥ 140/90 and on antihypertensive treatment	ATP
12	Mashinya et al., 2015 [37]	Cross sectional, South Africa, 2013–14	Both	44.8 ± 11.8	NA	NA	214	NA	≥ 140/90 and on antihypertensive treatment	ATP
13	Guira et al., 2016 [31]	Cross sectional, Burkina Faso, 2011	Both	44.8 + 7.4	NA	NA	300	NA	≥ 130/85 and on antihypertensive treatment	IDF
14	Hirigo et al., 2016 [36]	Cross sectional, Ethiopia, 2013	Both	26.5–38	NA	NA	185	NA	≥ 130/85 and on antihypertensive treatment	IDF, ATP
15	Zannou et al., 2009 [28]	Cohort, Benin, 2004–09	Both	38.0 ± 9.7	NA	NA	79	NA	≥ 130/85 and on antihypertensive treatment	IDF
16	Muyanja et al., 2016 [39]	Cross sectional, Uganda, NS	Both	30–43	NA	NA	250	NA	≥ 140/90 and on antihypertensive treatment	ATP
17	Adébayo et al., 2015 [44]	Cross-sectional, Benin, NS	Both	40,7 ± 9,71	NA	NA	244	NA	≥130/85 and on antihypertensive treatment	Other
18	Sawadogo et al., 2005 [43]	Cross sectional, Burkina Faso, 2011	Both	41.4 ± 8.8	NA	NA	400	NA	≥ 140/90 and on antihypertensive treatment	IDF, ATP

*NA* not applicable, *IDF* International Diabetes Federation, *ATP* Adult Treatment Panel III report of the National Cholesterol Education Program, *WHO* World Health Organization, *α* HIV negative

**Fig. 2:**
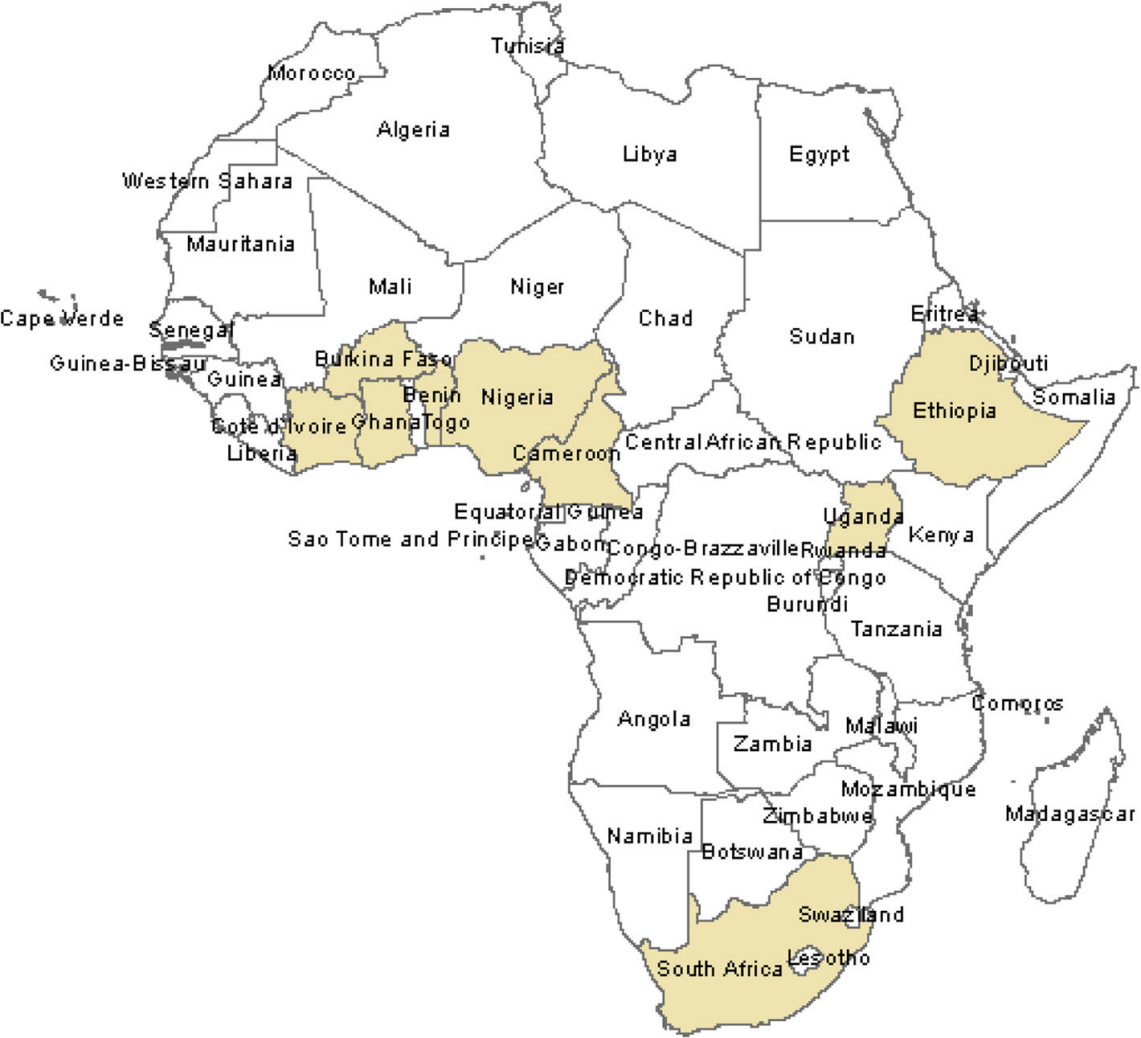
Map of Africa indicating the regions where the included studies were situated

Study breakdown from the three regions of sub-Saharan Africa were as follows: West Africa, 11; Southern Africa, 3, and Eastern Africa, 4. By countries, the distribution of studies were as follows: Nigeria, 3 [32, 33, 45]; Benin Republic, 2 [28, 44]; Burkina Faso, 2 [31, 43]; Cameroon, 2 [38, 40]; Ghana, 1 [41]; Ivory Coast, 1 [30]; South Africa, 3 [29, 35, 37]; Uganda, 1 [39]; and three studies from Ethiopia [34, 36, 42]. Of the four studies that presented estimates by HIV status, two were in Cameroon and one was in Nigeria and South Africa respectively Fig. 2.

Most of the studies (16 studies) were cross-sectionally designed [28, 29, 31–34, 36–45], with one randomized control trial and case-control study respectively [30, 35]. Most of the studies were hospital-based (16) with only two that were community-based. More than half (10 studies) were published after 2015–2016, and two studies were published in French [43, 44].

In the “Methods” section of the included studies, the projected sample sizes were bigger than the actual number of participants analyzed and reported. The number of participants presented in the studies analysis ranged between 50 to 300 HIV-negative participants and 79 to 755 participants in people living with HIV. About 25% of the total study participant’s samples were men, and the age of participants ranged from 18 to 70 years. In the 12 studies with data on the duration of ART, the duration ranged from 3 to 92 months.

The included studies applied various international criteria to diagnose MetS. Six studies defined metabolic syndrome using the International Diabetes Foundation (*IDF 2005*) criteria, four studies used the Third Report of the National Cholesterol Education Program and Expert Panel on Detection, Evaluation and Treatment of High Blood Cholesterol in Adults (Adult Treatment Panel III) (NCEP/ATP III) criteria, while four studies presented both NCEP/ATP III and *IDF 2005 criteria*. One study employed the *IDF 2005*, NCEP/ATP III, and JIS criteria and another with the *IDF 2005*, NCEP/ATP III, and WHO criteria. Two of the included studies did not clearly specify the definition employed. The included studies reported a variety of metabolic syndrome subcomponent [28, 30–40, 42–45] as described in Table 2.

**Table 2:** Qualitative description of metabolic syndrome subcomponents prevalence within included studies

	Author and publication year	Metabolic syndrome subcomponent	
		HIV+	HIV−
1	Amusa et al., 2016 [33]	Hypertension, diabetes, visceral obesity	Hypertension, diabetes, visceral obesity
2	Ayodele et al., 2012 [32]	Hypertension, diabetes, visceral obesity, high triglyceride, low HDL cholesterol	–
3	Berhane et al., 2012 [34]	Hypertension, diabetes, visceral obesity, high triglyceride	–
4	Tesfaye et al., 2014 [42]	Hypertension, diabetes, high triglyceride, low HDL cholesterol	–
5	Sobieszczyk et al., 2016 [29]	Diabetes, visceral obesity, high triglyceride, low HDL cholesterol	–
6	Obirikorang et al., 2016 [41]	–	–
7	Ngatchou et al., 2013 [40]	Diabetes	Diabetes
8	Fourie et al., 2010 [35]	Hypertension, diabetes, visceral obesity, high triglyceride, low HDL cholesterol	Hypertension, diabetes, visceral obesity, high triglyceride, low HDL cholesterol
9	Muhammad et al., 2013 [45]	Hypertension, diabetes, low HDL cholesterol	–
10	Mbunkah et al., 2014 [38]	Hypertension	–
11	Guehi et al., 2016 [30]	Hypertension, diabetes, visceral obesity, high triglyceride	–
12	Mashinya et al., 2015 [37]	Hypertension, diabetes, high triglyceride, low HDL cholesterol	–
13	Guira et al., 2016 [31]	Hypertension, diabetes, high triglyceride, low HDL cholesterol	–
14	Hirigo et al., 2016 [36]	Hypertension, diabetes	–
15	Zannou et al., 2009 [28]	Hypertension, diabetes, visceral obesity, high triglyceride	–
16	Muyanja et al., 2016 [39]	Hypertension, high triglyceride, low HDL cholesterol	–
17	Adébayo et al., 2015 [44]	Hypertension, diabetes, high triglyceride	Hypertension, diabetes, visceral obesity
18	Sawadogo et al., 2005 [43]	Diabetes	–

#### Risk of bias

A summary of the risk of bias of the included articles is shown in Appendix 1 and 2. Sixteen studies (88%) were considered to be at low risk of bias while the remaining two studies were classified as medium risk of bias. None of the studies was classified as high risk of bias. The major risks of bias were the lack of presentation of the representativeness of the study sample in relation to the broader target population (*n* = 8 studies, 44%) and the lack of specification of a random selection of subjects (*n* = 11 studies, 61%).

#### Quality assessment

A summary of the quality assessment of the included studies shows that half of the studies (*n* = 9, 50%) were considered to be of low quality, six studies were considered to be of medium quality, and three studies were considered to be of high quality. Most of the studies were cross-sectional in design, and this was deemed the weakest identified quality domain during our assessment due to the associated temporality bias.

#### Outcome measurement

##### Prevalence

The MetS prevalence estimates (Table 3) for the included studies ranged from the highest observed in south-western Uganda (145/250; 58.0%) [39] to the lowest in Abidjan (47/755; 6.23%) among people living with HIV populations [30]. Similarly, the prevalence estimates among HIV-uninfected population ranged from lowest in Nigeria (1/50; 2.0%) [33] to the highest in South Africa (68/300; 22.6%) [35]. The prevalence rate of the sub-components varies by presentation in the included studies (Table 4). Fourteen (77.8%) and 3 (75.0%) studies reported hypertension prevalence among people living with HIV and uninfected study participants respectively. Diabetes was reported among the 4 (100.0%) HIV-uninfected focused studies, while 15 (83.3%) infected population studies. High triglycerides were reported in 12 (67.0%) studies among infected population while 1 (25.0%) uninfected population study. Visceral obesity prevalence was presented in 7 (39.0%) studies among infected cohorts, and 2 (50.0%) studies reported it among uninfected cohorts. Similarly, 7 (39.0%) studies among the infected participants reported low HDL cholesterol as against 1 (25.0%) study in the uninfected cohorts.

**Table 3:** Prevalence of MetS by definition

	Author and publication year	HIV status		Prevalence by definition criteria		
		HIV+	HIV−	IDF	ATP	Others
1	Amusa et al., 2016 [33]	150	50	NA	NA	41 (27.3%), *P* < 0.01^Ϯ^ 2 (4%), *P* < 0.01^α^—not stated
2	Ayodele et al., 2012 [32]	291	NA	50 (17.2%)	37 (12.7%)	61(21.0%)—JIS
3	Berhane et al., 2012 [34]	313	NA	NA	66 (21.1%)	NA
4	Tesfaye et al., 2014 [42]	374	NA	23.8%	16.8%	NA
5	Sobieszczyk et al., 2016 [29]	160	NA	NA	27 (8.7%)	NA
6	Obirikorang et al., 2016 [41]	433	NA	183 (42.3%)	209 (48.3%)	106 (24.5%)—WHO
7	Ngatchou et al., 2013 [40]	108	96	47.0%, *P* = 0.02^Ϯ^ 21.0%, *P* = 0.02^α^	NA	NA
8	Fourie et al., 2010 [35]	300	300	21.1%, *P* = 0.65^Ϯ^ 22.6%, *P* = 0.65^α^	15.2% *P* = 0.18^Ϯ^ 11.5% *P* = 0.18^α^	NA
9	Muhammad et al., 2013 [45]	200	NA	ART = 21.0%; Naive = 9.0% *P* = 0.017	NA	NA
10	Mbunkah et al., 2014 [38]	173	50	NA	15.6% (27/173) (*P* = 0.020)^Ϯ^ 8.0% (4/50)^α^	NA
11	Guehi et al., 2016 [30]	755	NA	NA	47 (6.2%) *P* > 0.0001	NA
12	Mashinya et al., 2015 [37]	214	NA	NA	20 (9.6%) *P* = 0.56	NA
13	Guira et al., 2016 [31]	300	NA	54 (18.0%)	NA	NA
14	Hirigo et al., 2016 [36]	185	NA	24.3% (45/185)	17.8%	NA
15	Zannou et al., 2009 [28]	79	NA	10 (12.7%)	NA	NA
16	Muyanja et al., 2016 [39]	250	NA	NA	145/250 (58.0%) *P* value = 0.10	NA

,*NA* not applicable

^α^HIV-negative

^Ϯ^HIV-positive

**Table 4:** Qualitative description of metabolic syndrome subcomponents prevalence within included studies

Author and publication year		Hypertension		Diabetes		Visceral obesity		High triglycerides		Low HDL cholesterol	
		HIV+	HIV−	HIV+	HIV−	HIV+	HIV−	HIV+	HIV−	HIV+	HIV−
1	Amusa et al., 2016 [33]	46.0% *P* < 0.01	5/50 (10.0%) *P* < 0.01	42/150 (28.0%) *P* < 0.01	2/50 (4.0%) *P* < 0.01	48/150 (32.0%) *P* 0.79	15/50 (30%) *P* 0.79 (*P* = 0.79)	NP	NP	NP	NP
2	Ayodele et al., 2012 [32]	82 (28.2%), *P* = 0.146	NP	54 (18.6%) *P* = 0.600	NP	56 (19.2%) *P* < 0.001	NP	38 (13.1%) *P* = 0.880	NP	159 (54.6) *P* = 0.013	NP
3	Berhane et al., 2012 [34]	110/313 (35.1%)	NP	78/313 (24.9%)		43/313 (13.7%)		83/31 (26.5%)			NP
4	Tesfaye et al., 2014 [42]	SBP = 39/374 DBP = 33/374	NP	103	NP	NP	NP	154	NP	248	NP
5	Sobieszczyk et al., 2016 [29]	NP	NP	(0.7 to 1.9%) *P* = 0.346		33.5 to 44.3% (*P* = 0.060)		9.4 to 13.3%, *P* = 0.112		56.6 to 61.0%, *P* = 0.283	
6	Obirikorang et al., 2016 [41]	NP	NP	NP	NP	NP	NP	NP	NP	NP	NP
7	Ngatchou et al., 2013 [40]	NP	NP	26% *P* < 0.01	1% *P* < 0.01	NP	NP	NP	NP	NP	NP
8	Fourie et al., 2010 [35]	50.0% *P* = 0.03	59.0% *P* = 0.03	ATP III 22.7% *P* = 0.49 IDF 36.6% *P* = 0.08	ATP III 25.1% *P* = 0.49 IDF 43.7% *P* = 0.08	ATP III Male—0.9% *P* = 0.32 Female—18.3% *P* = 0.93 IDF Male—2.6% *P* = 0.31 Female—33.9% *P* - 0.22 (*P* = 0.22)	ATP III Male—0.0% *P* - 0.32 (*P* = 0.32) Female—18.7% *P* - 0.93 (*P* = 0.93) IDF Male—0.9% *P* - 0.31 (*P* = 0.31) Female—40.1% *P* - 0.22 (P = 0.22)	ATP III 18.2% *P* = 0.19 IDF 14.3% *P* = 0.19	ATP III 17.6% *P* = 0.28 IDF 14.3% *P* = 0.28	ATP III Male—47.4%, Female—62.6% *P* < 0.0001 IDF Male—46.5% *P* < 0.0001 Female—62.6% *P* < 0.0001	ATP III Male—12.1% *P* < 0.0001 Female—33.7% *P* < 0.0001 IDF Male—11.2% *P* < 0.0001 Female—33.7% *P* < 0.0001
9	Muhammad et al., 2013 [45]	9.5 (*P* < 0.001).	NP	3 (*P* = 1.0)	NP	NP	NP	16		68.5%	
10	Mbunkah et al., 2014 [38]	24.7%	NP	NP	NP	NP	NP	NP	NP	NP	NP
11	Guehi et al., 2016 [30]	37 (4.9%)	NP	4 (0.5%)	NP	128 (17.0%)	NP	128 (17.0%)	NP	NP	NP
12	Mashinya et al., 2015 [37]	56 (26.2%)	NP	10 (4.7%)	NP	NP	NP	Male = 35.0 vs female = 12.5%, *P* = 0.001)		91 (43.8%)	
13	Guira et al., 2016 [31]	36 (66.7%)	NP	16 (29.6%)				27 (50%)		37 (68.5%)	
14	Hirigo et al., 2016 [36]	18/185 *P* = 0.84	NP	IDF criteria 58 (31.3%)	NP	NP	NP	NP	NP	NP	NP
15	Zannou et al., 2009 [28]	29 (42.6)		6 (7.6%)		24 (33.3%)	NP	10 (14.1%)	NP	NP	NP
16	Muyanja et al., 2016 [39]	13 (5.2%) *P* = 0.46	NP	NP	NP	NP	NP	74 (29.6%) 0.76	NP	214 (85.6%) 0.16	NP
17	Adébayo et al., 2015 [44]*	60 (24.6%)	5 (10%), *P* < 0.01	5 (2.04%)	2 (4.0%)	NP	NP	44 (18.0%) Male—12 (12.6%) Female—26 (13.3%)	NP	NP	NP
18	Sawadogo et al., 2005 [43]*	NP	NP	1.3%, CI (0.5–3.0)	NP	NP	NP	NP	NP	NP	NP

*French publication

##### Sex variation

Seven studies [28, 31, 32, 34, 36, 37, 39] presented estimates of MetS stratified by sex among people living with HIV subjects. The prevalence estimates in males ranged from (6/95; 6.3%) [32] in Nigeria to (41/81; 50.6%) [39] in Uganda using ATP III definition. Similarly, the estimated prevalence using IDF definition among males ranged from (1/38; 3.1%) [28] in Benin to (8/93;14.9%) [31] in Burkina Faso. Among females, MetS estimate ranged from (9/38; 19.2% [28] and 46/207; 85.1% [31]) to (15/171; 8.9% [37] and 104/169; 61.5% [39]) using IDF and ATPIII definition respectively.

Notable, MetS were shown to be consistently more prevalent in female than male across the two criteria. There is a relative estimate of 12.7% was among the female against 3.6% using IDF criteria, and ATP III definition estimated MetS prevalence at 19.7% among females and 15.7% in males respectively. The sex variation in MetS prevalence among HIV-negative cohort could not be ascertained because it was not reported.

##### Meta-weighted prevalence of MetS

**Fig. 3:**
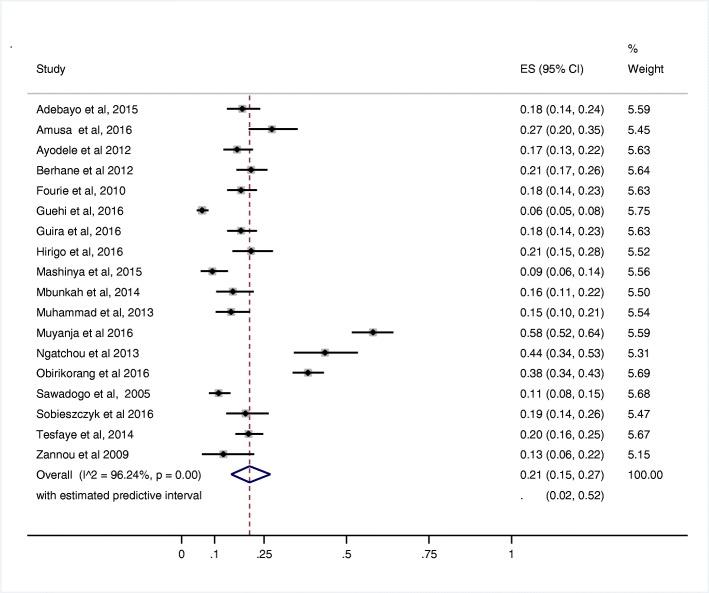
Forest plot of the prevalence of metabolic syndrome in studies on HIV-positive subjects

**Fig. 4:**
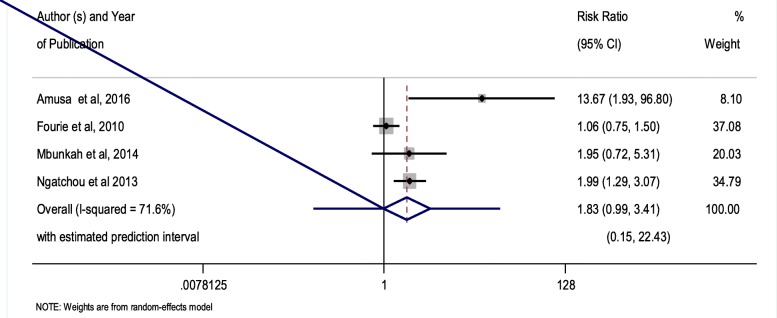
Forest plot of the prevalence ratios of metabolic syndrome comparing HIV-positive to HIV-negative subjects

The meta-prevalence of MetS measured among people living with HIV subjects irrespective of the MetS definition employed was 21.5% (95% CI 16.09–26.86%)—Fig. 3. The prevalence of MetS among HIV-positive population measured by IDF was higher than those measured by the ATP III definition at 25.7% (95% CI 16.62–34.79%) versus 19.9% (95% CI 12.26–27.45). Similarly, the meta-prevalence of the MetS in HIV-negative subjects in this review was 12.0% (95% CI 5–21%). The overall relative risk of MetS prevalence among people living with HIV population compared with HIV-uninfected population was 1.83 (95% CI 0.99–3.41), with an estimated predictive interval of (0.15 to 22.43) *P* value = 0.055—Fig. 4.

##### Publication bias

A funnel plot assessing the pooled prevalence of metabolic syndrome among people living with HIV populations suggested a weak publication bias among the included studies. Egger test results (*P* = 0.271) did not indicate significant small study effect bias when considering HIV-positive studies. Also, a random effects variance shift outlier model analysis suggested that the Muyanja et al. [39] study was a prominent and influential outlier in our study. Thus, further meta-regression analysis to identify the source of heterogeneity was performed and also suggests that risk of bias score and year were potential sources of heterogeneity. However, a meta-regression model including year and risk of bias score and excluding the aforementioned influential outlier study only reduced the adjusted *I*^2^ to ~ 65%. Hence, there was still a large residual heterogeneity which is a potential limitation of the underlying data.

## Discussion

### MetS prevalence overview

To our knowledge, this is the first systematic review and meta-analysis of the MetS prevalence in the sub-Saharan African population by HIV status. Notably, the total prevalence of MetS among people living with HIV population was significantly higher at 21.5% (95% CI 15.09 to 26.86) with estimated predictive interval of (0.02 to 0.52) irrespective of the definition criteria compared with their HIV-negative counterparts at 12.0% (95% CI 5–21%). However, the wide prevalence range indicates substantial heterogeneities and this is as a result of influential outlying estimates from one of the included study.

As mentioned above this review suggests a twofold higher risk of MetS (RR 1.83, 95% CI 0.98–3.41), with an estimated predictive interval of (0.15 to 22.43) *P* value = 0.055 among people living with HIV subjects compared to their HIV-negative counterparts, and this ratio was not statistically significant. This finding suggests HIV infection and ART appear to contribute to a significant excess burden of MetS over and above the contribution of traditional lifestyle-related risk factors. The findings of this review are somewhat related to ones discussed in other studies [46–48]. The meta-prevalence of MetS among people living with HIV populations in SSA countries has shown to be higher when compared to reported estimates from developed countries [18, 49]. Similarly, the estimate of MetS among the uninfected population in this review is similar to the AGEhIV cohort study in the Netherlands, underpinning the significance of excess MetS risk among people living with HIV compared to HIV-negative [50]. Irrespective of the risk differences of MetS among people living with HIV and uninfected patients in this review not being significant, it is notable that the prevalence of MetS in both cohorts are high and ranged within the earmarked global burden. This implies that the burden of MetS is growing vehemently in SSA with or without HIV; hence, other related factors such as lifestyles, diets, aging, and other interlinked factors require crucial prevention and management beyond HIV.

Across the major criteria (ATP 2001, IDF 2005, JIS) used by most studies included in this review, the estimated MetS prevalence was highest based on the IDF 2005 definition (25.7%). This was different from a similar review that found higher estimates based on the ATP definition criteria [12]. This implies a large waist circumference band among the infected cohorts included in this review, especially females, as this is a compulsory requirement using IDF definition. This agrees with the finding underlining high adiposity (based on body mass index) and waist circumference among people living with HIV subjects [18, 51]. Further research is needed to understand the difference between waist circumferences by HIV status, as this was not established in this review.

The analysis of the meta-prevalence of MetS individual subcomponents was limited given the lack of adequate reporting in the included studies. Diabetes and hypertension were the most reported sub-components, but among people living with HIV cohorts. Nonetheless, studies have described the outcome of the high prevalence of hypertension and diabetes among people living with HIV populations [52, 53]. Further research is required to understand which of the sub-component is the most prevalent and contributes to the development of MetS by HIV status.

With the widespread ART uptake and the introduction of treat, all strategy irrespective of viral load and CD4 count, we anticipated an increase in the burden of MetS irrespective of age and sex in SSA. Despite an unbalance representation of people living with HIV studies against uninfected groups in this review, the prevalence of MetS was essentially higher among the infected population. The absence of study conducted specifically on populations with confirmed negative HIV status might have accounted to these findings. Otherwise, it may be the true reflection of the ubiquitous MetS burden in sub-Saharan Africa irrespective of HIV status, as a result of improved life expectancy, globalization, and lifestyles. Thus, a scale-up in the awareness, prevention, and management of metabolic disorder is directly needed in this continent, to curb the emerging epidemic.

## Strengths and limitations

### Strengths

This review strictly adhered to the PRISMA guidelines to maximize the robustness and rigor of the employed methodology. We also conducted a very rigorous quality and risk of bias assessment. To our knowledge, this is the first systematic review to attempt to compare the burden of MetS by HIV status. Furthermore, we did not exclude French-based studies given the region of focus in this review and a large number of Francophile countries with HIV burden.

### Limitations

Our findings may not be generalizable to all people living with HIV and uninfected individuals given the small number of included studies and potential non-representativeness. Given the varied methodological designs of the studies included in the review, the calculation of the pooled prevalence estimate may have been affected by this heterogeneity, as suggested by the very high *I*^2^ statistic of 96.05% based on the final eligible pool of studies included in this meta-analysis. Some of the included study’s authors were contacted for raw data and further clarifications; however, some estimates had to be calculated from data provided in the article. One of the included studies among people living with HIV cohort was a randomized control trial study, but we do not suspect that the inclusion and exclusion criteria of the trial participants would introduce substantial bias to our study, as these are not related to the metabolic risk factors/conditions we are attempting to assess among adults living with HIV. The pooled risk ratio of the burden of metabolic syndrome among HIV-infected population compared to their negative counterparts should be interpreted with caution given that only 4 out of 18 studies compared MetS by HIV status. Furthermore, limited comparison of pooled prevalence of MetS combining all 18 studies versus meta-estimate among combined HIV-negative subjects in the 4 studies (and relatively small pooled sample size of HIV-negative subjects) can be done as the underlying epidemic profile varies tremendously across the region. A further limitation was the lack of standardized MetS definition employed across the various study settings.

Another limitation of this review was the limited subgroup stratification of MetS burden by sex and ART regimen among the eligible studies. However, the association of antiretroviral therapy with MetS has been previously documented [12, 54]. Also, the relationship between the use of ART and naïve with MetS prevalence could not be ascertained in this review, as 16 out of 18 studies on HIV-infected subjects reported that the participants were ART-experienced. This may explain the high prevalence of MetS quantified in this review. It is thus important to include routine and regular metabolic disorder check in the routine follow-ups of people living with HIV to optimize prevention and management, especially in the era of treat-all.

Thus, further research is required in estimating the extent and association of HIV status and metabolic syndrome and/or its subcomponents within SSA. Moreover, a more standardized approach of classifying MetS in SSA should be adopted to allow better comparability across countries in the region and whether conventional waist circumference cutoffs are appropriate in the Africa context.

## Conclusion and implications

MetS prevalence in people living with HIV and uninfected individuals is high in sub-Saharan Africa; however, based on our findings, this appears to be a non-significant high prevalence comparing HIV-positive and HIV-negative group. However, this review needs to be interpreted with caution given the weaknesses alluded to above. More primary research is required in SSA to give a better understanding of the difference in the burden of metabolic syndrome in the context of high HIV burden. However, the findings of this review have implications for public health practice and policymakers within SSA as HIV-positive individual’s life expectancy increases in the post ART rollout era and also in the context of an unfolding epidemiological transition where an increasing burden of non-communicable in the context of a high dual and persistent burden of infectious disease. Implementing an inclusive/integrated care plan for people living with HIV populations in the region is essential. This implies the increased presence of other healthcare needs beyond HIV and other communicable infection that might overburden our already overstrained healthcare systems. Early screening of metabolic syndrome subcomponents irrespective of HIV status to reduce future metabolic syndrome epidemic cost is important in the era of increased population aging and obesity, and this has been observed in SSA.

## Appendix 1

**Table 5:** Search strategy with MeSH terms

Search	Search terms	Number of hits PubMed	Number of hits Ebscohost	Number of hits Cochrane Database	Number of hits Web of Science
#1	metabolic syndrome OR syndrome X OR insulin resistance syndrome	187,904	27,587	10,928	128,787
#2	Hypertension OR high blood pressure	533723	102,771	89,556	107.083
#3	Type 2 diabetes mellitus OR type 2 diabetes OR diabetes Mellitus OR non-insulin dependent diabetes OR adult onset diabetes	435357	92,546	38,353	377,811
#4	Human Immunodeficiency Virus OR Acquired Immune Deficiency Syndrome Virus OR AIDS Virus OR HIV Seronegativities OR Seronegativity, HIV OR HIV Seropositivities OR Seropositivity, HIV	329085	139,326	8285	268,598
#5	#1 OR #2 OR #3 AND #4	8693	137,326	97,517	134,466
#6	African filter((((Angola OR Benin OR Botswana OR “Burkina Faso” OR Burundi OR Cameroon OR “Cape Verde” OR “Central African Republic” OR Chad OR Comoros OR Congo OR “Democratic Republic of Congo” OR Djibouti OR “Equatorial Guinea” OR Eritrea OR Ethiopia OR Gabon OR Gambia OR Ghana OR Guinea OR “Guinea Bissau” OR “Ivory Coast” OR “Cote d’Ivoire” OR Kenya OR Lesotho OR Liberia OR Madagascar OR Malawi OR Mali OR Mauritania OR Mauritius OR Mozambique OR Namibia OR Niger OR Nigeria OR Principe OR Reunion OR Rwanda OR “Sao Tome” OR Senegal OR Seychelles OR “Sierra Leone” OR Somalia OR “South Africa” OR Sudan OR Swaziland OR Tanzania OR Togo OR Uganda OR “Western Sahara” OR Zambia OR Zimbabwe OR “Central Africa” OR “Central African” OR “West Africa” OR “West African” OR “Western Africa” OR “Western African” OR “East Africa” OR “East African” OR “Eastern Africa” OR “Eastern African” OR “South African” OR “Southern Africa” OR “Southern African” OR “sub Saharan Africa” OR “sub Saharan African” OR “sub Saharan Africa” OR “sub Saharan African” NOT “guinea pig” NOT “guinea pigs” NOT “aspergillus niger” ))))	310426	354,204	15,628	467,826
#7	# 5 AND # 6 Limits: 01/01/1990 to 28/02/2017 in English and French on humans	632	7960	1825	160
Total = 125	Title screening	98	25	0	2

## Appendix 2

**Table 6:** Presentation of the risk of bias of included studies

S/*N*	Author (s) and year of publication	Was the study’s target population a close representation of the national population in relation to relevant variables, e.g. age, sex, occupation?	Was the sampling frame a true or close representation of the target population?	Was some form of random selection used to select the sample, OR, was a census undertaken?	Was the likelihood of non-response bias minimal?	Were data collected directly from the subjects (as opposed to a proxy)?	Was an acceptable case definition used in the study?	Was the study instrument that measured the parameter of interest (e.g. prevalence of low back pain) shown to have reliability and validity (if necessary)?	Was the same mode of data collection used for all subjects?	Were the numerator(s) and denominator r(s) for the parameter of interest appropriate	Summary on the overall risk of study bias
1	Adébayo et al., 2015 [44]	No (high risk)	No (high risk)	No (high risk)	No (high risk)	Yes (low risk)	Yes (low risk)	Yes (low risk)	Yes (low risk)	Yes (low risk)	Moderate risk (4–6)
2	Amusa et al., 2016 [33]	Yes (low risk)	No (high risk)	No (high risk)	No (high risk)	Yes (low risk)	Yes (low risk)	Yes (low risk)	Yes (low risk)	Yes (low risk)	Moderate risk (4–6)
3	Ayodele et al., 2012 [32]	Yes (low risk)	Yes (low risk)	No (high risk)	Yes (low risk)	Yes (low risk)	Yes (low risk)	Yes (low risk)	Yes (low risk)	Yes (low risk)	Low risk (0–3)
4	Berhane et al., 2012 [34]	Yes (low risk)	Yes (low risk)	No (high risk)	No (high risk)	Yes (low risk)	Yes (low risk)	Yes (low risk)	Yes (low risk)	Yes (low risk)	Low risk (0–3)
5	Fourie et al., 2010 [35]	Yes (low risk)	Yes (low risk)	Yes (low risk)	Yes (low risk)	Yes (low risk)	Yes (low risk)	Yes (low risk)	Yes (low risk)	Yes (low risk)	Low risk (0–3)
6	Guehi et al., 2016 [30]	Yes (low risk)	Yes (low risk)	Yes (low risk)	Yes (low risk)	Yes (low risk)	Yes (low risk)	Yes (low risk)	Yes (low risk)	Yes (low risk)	Low risk (0–3)
7	Guira et al., 2016 [31]	No (high risk)	No (high risk)	No (high risk)	Yes (low risk)	Yes (low risk)	Yes (low risk)	Yes (low risk)	Yes (low risk)	Yes (low risk)	Low risk (0–3)
8	Hirigo et al., 2016 [36]	No (high risk)	No (high risk)	No (high risk)	Yes (low risk)	Yes (low risk)	Yes (low risk)	Yes (low risk)	Yes (low risk)	Yes (low risk)	Low risk (0–3)
9	Mashinya et al., 2015 [37]	No (high risk)	Yes (low risk)	No (high risk)	Yes (low risk)	Yes (low risk)	Yes (low risk)	Yes (low risk)	Yes (low risk)	Yes (low risk)	Low risk (0–3)
10	Mbunkah et al., 2014 [38]	No (high risk)	Yes (low risk)	Yes (low risk)	Yes (low risk)	Yes (low risk)	Yes (low risk)	Yes (low risk)	Yes (low risk)	Yes (low risk)	Low risk (0–3)
11	Muhammad et al., 2013 [45]	Yes (low risk)	Yes (low risk)	Yes (low risk)	Yes (low risk)	Yes (low risk)	Yes (low risk)	Yes (low risk)	Yes (low risk)	Yes (low risk)	Low risk (0–3)
12	Muyanja et al., 2016 [39]	No (high risk)	No (high risk)	No (high risk)	Yes (low risk)	Yes (low risk)	Yes (low risk)	Yes (low risk)	Yes (low risk)	Yes (low risk)	Low risk (0–3)
13	Ngatchou et al., 2013 [40]	Yes (low risk)	Yes (low risk)	No (high risk)	No (high risk)	Yes (low risk)	Yes (low risk)	Yes (low risk)	Yes (low risk)	Yes (low risk)	Low risk (0–3)
14	Obirikorang et al. 2016 [41]	Yes (low risk)	Yes (low risk)	No (high risk)	No (high risk)	Yes (low risk)	Yes (low risk)	Yes (low risk)	Yes (low risk)	Yes (low risk)	Low risk (0–3)
15	Sawadogo et al., 2005 [43]	Yes (Low risk)	Yes (low risk)	Yes (low risk)	No (high risk)	Yes (low risk)	Yes (low risk)	Yes (low risk)	Yes (low risk)	Yes (low risk)	Low risk (0–3)
16	Sobieszczyk et al., 2016 [29]	Yes (low risk)	Yes (low risk)	No (high risk)	Yes (low risk)	Yes (low risk)	Yes (low risk)	Yes (low risk)	Yes (low risk)	Yes (low risk)	Low risk (0–3)
17	Tesfaye et al., 2014 [42]	No (high risk)	Yes (low risk)	Yes (low risk)	Yes (low risk)	Yes (low risk)	Yes (low risk)	No (high risk)	Yes (low risk)	Yes (low risk)	Low risk (0–3)
18	Zannou et al., 2009 [28]	No (high risk)	Yes (low risk)	Yes (low risk)	Yes (low risk)	Yes (low risk)	Yes (low risk)	Yes (low risk)	Yes (low risk)	Yes (low risk)	Low risk (0–3)

## Abbreviations

ART: Antiretroviral therapy
ATP III/NCEP: Adult Treatment Panel III report of the National Cholesterol Education Program
HIV+: Human immunodeficiency virus-positive
HIV/AIDS: Human immunodeficiency virus and acquired immune deficiency syndrome
HIV−: Human immunodeficiency virus-negative
IDF: International Diabetes Federation
MetS: Metabolic syndrome
NA: Not applicable
NCD: Non-communicable diseases
NP: Not presented
